# Common mental disorders and associated factors among mothers of children attending severe acute malnutrition treatment in Gedio Zone, Southern Ethiopia, 2022: a cross-sectional study

**DOI:** 10.1186/s12888-024-05741-z

**Published:** 2024-04-12

**Authors:** Bekahegn Girma, Kirubel Bimer, Chalachew Kassaw, Nebiyu Mengistu, Ashenafi Zewdie, Jerusalem Sewalem, Derebe Madoro

**Affiliations:** 1 Department of Nursing, College of Health Sciences and Medicine, Dilla University, Dilla, Ethiopia; 2 Department of Psychiatry, College of Health Science and Medicine, Dilla University, Dilla, Ethiopia

**Keywords:** Common mental disorders, Malnutrition, Gedio, Ethiopia

## Abstract

**Background:**

Common mental disorders describe the physical, mental, and social disturbances that are more prevalent in low and middle-income countries. Mothers are among the more vulnerable groups especially mothers having children with under-nutrition. However, there are limited studies about the magnitude of common mental disorders among mothers of undernourished children in Ethiopia. Therefore, we aimed to assess the magnitude of common mental disorders and associated factors among mothers of children attending severe acute malnutrition treatment in Gedio Zone, Southern Ethiopia.

**Methods:**

A cross-sectional study was employed on 405 systematically selected participants. The outcome variable was assessed by a self-reporting questionnaire (SRQ-20) which was applicable and validated in Ethiopia. Data were entered and analyzed by EPi data version 5 software and SPSS version 25 respectively. Model fitness was checked by Hosmer Lemeshow’s test. Logistic regression was employed to identify significant determinants. A p-value < 0.05 was used to declare association and expressed by odds ratio with a 95% CI.

**Result:**

In this study, the magnitude of common mental disorders was 33.16% (95% CI [28.5–38])). In multivariable analysis, six factors poor social support [AOR: 14.0, 95% CI (5.45, 35.9)], educational status [AOR: 1.95, 95% CI (1.07. 3.55)], cigarette smoking [AOR: 10.9, 95% CI (1.78, 67.01)], mother of a child with another chronic disease [AOR: 3.19, 95% CI (1.13, 8.99)], sexual violence [AOR: 4.14, 95% CI (1.38, 12.4)] and mothers with chronic disease [AOR: 3.44, 95% CI (1.72, 6.86)] were significantly associated with common mental disorders.

**Conclusion:**

The magnitude of common mental disorders was high. Six factors were significantly associated with common mental disorders; social support, sexual violence, maternal chronic illness, educational status, smoking, and mother of child with other chronic disease. Community awareness regarding the effect of violence, substance use, and social support on mental health should be created by the local stakeholders.

## Introduction

Common mental disorder (CMD) is a gross name for physical, mental, and social disturbances used to describe a range of symptoms like depression, anxiety, or somatic manifestations [1]. It has adverse economic, social and psychological consequences for the individuals, their children, their families and the community [2].

Globally, 4.4% of mothers of malnourished children had CMD. Studies from different countries showed that the prevalence of CMD among mothers having children with under-nutrition is higher than mothers of children with normal nutritional. For instance, in Uganda and Kenya among mothers of malnourished children 42% and 64.1% had common mental disorder [3, 4]. Moreover, in Nigeria 40.7% of mothers of malnourished children had CMD [5].

A disproportionate number of common mental disorders (CMDs) are experienced by mothers of malnourished children in poor nations [6], 30–40% of this population are reported to have CMDs, according to studies, which is much greater than the 10–17.6% prevalence seen in the overall population [7–10].

Poor maternal mental health can adversely affect the nutrition, health, and psychological well-being of the child [4]. It also increases the risk of growth failure [11, 12]. CMD can disrupt the mothers’ ability to cope with the demands of childcare [13–15]. Reduction in the burden of CMD leads to a reduction in child growth retardation by 30%. Therefore, by doing this, it is possible to decrease both maternal and under-five mortality [16].

Mothers of malnourished children are more likely to experience common mental disorders due to a number of factors, such as poverty, food insecurity, educational attainment, stigma, lack of social support, mother’s nutritional status, quality of care, and limited access to healthcare. Moreover, the emotional strain of witnessing a child’s suffering from malnutrition can significantly contribute to the development of mental health issues in mothers [5, 17–19].

In 2014, 56% of under-five mortality was related to the exacerbating effects of malnutrition [20] and still now the mortality is high. Even though maternal CMD and SAM among children being strongly related and having a bi-directional relationship, maternal mental health is largely neglected in developing countries [21]. Furthermore, the magnitude and effect of maternal mental disorder on children nutritional status is unknown [22].

In Ethiopia, little is known about the magnitude of CMD among mothers’ of malnourished children. Therefore, we aimed to assess the magnitude of common mental disorders and associated factors among mothers of children attending SAM treatment in Gedio Zone, Southern Ethiopia, 2022.

## Methods and materials

### Study area and period

Our study was conducted in Gedeo zone public health centers. Gedeo zone is found in the South Nation Nationalities and People Region (SNNPR), and the capital city is Dilla, 394 Km away from Addis Ababa, which is the capital city of Ethiopia and 84 km away from Hawassa. The altitude of Dilla is 1765 m above sea level. Gedeo zone has a total population of 847,434; of whom 424,472 are males and 422,696 are female. Only 12.72% of the population lives in the urban areas. In this zone, the average household size of the family is 5.1. The livelihood of the population is also depending on agriculture and livestock production. Gedeo zone has a total of seven woredas and two administrative towns [23]. Our study was conducted in 3 woredas (Yirgachefe, Wonago and Bule) and one administrative town (Yirgachefe).

The medical facilities provide promotional, curative, rehabilitative, and preventative therapies to the local population. Women who are going through the postpartum period, giving birth, and attending prenatal check-ups are frequently given health education by the health clinics. But they didn’t give attention to maternal mental health of SAM children. The study was conducted from March 1, 2022 to April 30, 2022.

### Study design

Institution-based cross-sectional study design was employed on mothers of children (6–59 months) who are attending SAM treatment at an outpatient program.

### Population

#### Source population

All mothers of children (6–59 months) who are attending SAM treatment at an outpatient programs in Gedio Zone.

#### Study population

All mothers of children (6–59 months) who are attending SAM treatment at an outpatient program in the selected areas (Yirgachefe, Wonago, and Bule Woredas, and Yirgachefe town).

### Eligibility criteria

#### Inclusion criteria

All mothers-to-child (6–59 months) pairs who are attending SAM treatment at outpatient program in Gedio Zone were included.

### Sample size determination and sampling technique02

A single population proportion formula was used to calculate the sample size by considering the following statistical assumptions; P: proportion of maternal CMD in children attending health facilities in Nigeria which was 40.7% [5], Z_α/2_: the corresponding Z score of 95% CI, d: Margin of error (5%) and N: Sample size.

$${\text{N}}\,=\,\frac{{\left( {{\text{Z}}\alpha 2} \right)2\, \times \,{\text{p}}\,\left( {1\, - \,{\text{p}}} \right)}}{{\left( {\text{d}} \right)2}}$$

N: (1.96)2 *0.4*0.6/ (0.05)^2^: 369 then after adding a 10% non-response rate the final sample size was 405. A systematic random sampling method was used to select participants.

### Sampling procedure

**Fig. 1:**
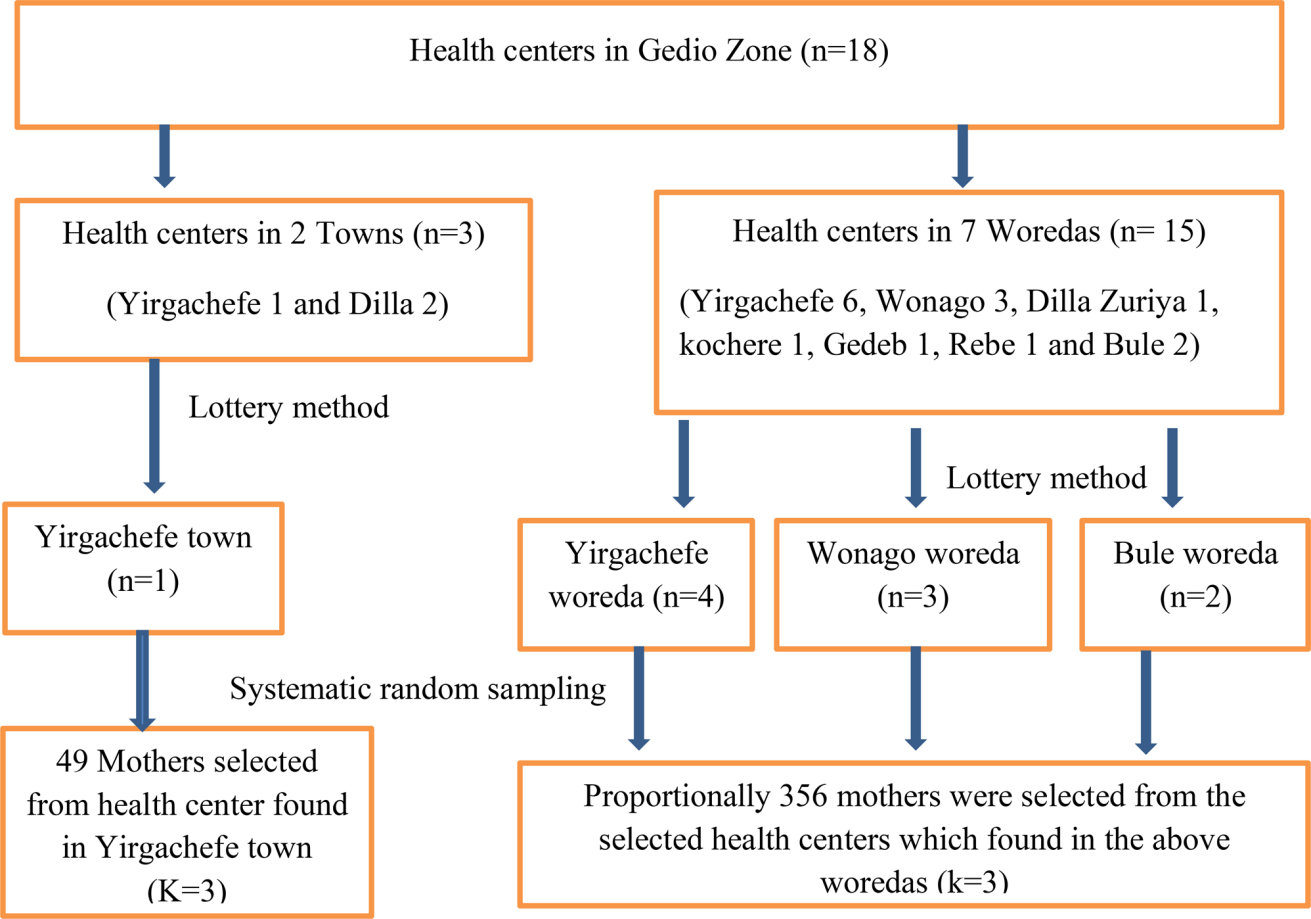
Schematic presentation of sampling procedure

Gedeo zone has a total of seven woredas and two administrative towns. In those woredas and administrative towns, there are 18 health centers. Our study was conducted in 3 woredas (Yirgachefe, Bule, and Wonago) and one administrative town (Yirgachefe) which was selected using lottery method. From Yirgachefe, Bule, Wonago woredas and Yirgachefe town 4, 2, 3, & 1 health centers were selected respectively. The sample size was proportionally allocated to the selected health centers based on patient follow. The medical record number of the children was used as a sampling frame. A systematic random sampling method was used to select each participant by using a K value of 3 (Fig. 1).

### Data collection tool and procedure

Data were collected using interviewer administered-structured questionnaire, which was adopted from previous studies and then adapted to the local contexts. The questionnaire includes information about socio-demographic, maternal and childhood-related characteristics.

The SRQ-20 was used to assess the dependent variable which was developed by the World Health Organization (WHO) as a screening tool. The tool was validated in multiple developing countries’ settings and has good sensitivity and specificity. It was a scale-based score consisting of 20 yes or no questions which ask about multiple symptoms which have occurred within the preceding 30 days. When a symptom was present within the specified period, the individual was given a score of 1, while 0 represents the absence of symptoms within the same period. The sum of all the scores represents the overall SRQ-20 score and higher scores represent poorer maternal mental health. We considered a cut-off maternal SRQ score of ≥ 8 to define maternal CMD which was based on previously published works [24]. Moreover, the Oslo Social Support scale was used to assess social support.

Ten BSc nurses who can speak Gede-uffa and Amharic were considered as data collectors. Along with them, two supervisors were involved during data collection time to supervise the overall data collection procedures. After signing a consent form, each participant was interviewed face-to-face.

### Operational definitions

#### Maternal CMD

is defined as those mothers who score ≥ 8 on SRQ scale [24].

#### Poor social support

expressed as those mothers scored 3–8 from the total sum score of 14 on Oslo Social Support scale [25].

#### Current substance use

described as mother who uses (non-medical use only) at least anyone of substance (alcohol, chat, or cigarette) currently or for the past three months [26].

#### Past and current medical illness

used to describe those mothers who have known chronic medical illness and their diagnosis confirmed in any health institution and currently have follow-up.

### Data quality assurance

The questionnaire was first developed in English version then translated in to *Gede-uffa* and Amharic languages. To ensure its consistency, it was translated back into English. The interview was conducted using a Gede-uffa version questionnaire. Two days of training were given to data collectors and supervisors; about the purpose, study tool and overall data collection procedure to be eminently maintained by them during data collection time. Pre-test was conducted on 5% of the study participants.

### Data analysis

The data were checked for completeness and consistency. It was entered and analyzed by EPi data version 5 software and SPSS version 25 respectively. Descriptive statistics were used to explain the study participant’s data. The model fitness was checked by Hosmer Lemeshow’s test. Bivariate and multivariable logistic regression analysis was conducted to identify the associated factors. Those variables with a p-value < 0.25 on bivariate analysis were selected for multiple logistic regression. A P-value less than 0.05 were considered statistically significant and an association was expressed by odds ratio with a 95% Confidence interval.

## Result

Among the total of 405 participants, only 389 were eligible with a response rate of 96.04%.

### Socio-demographic characteristics

One hundred thirty-six (35%) of mothers were in the age group of 25–29. Two hundred eighty-eight (74%) and three hundred eleven (79.9%) of mothers were protestant and married. Three hundred twenty-eight (84.3%) were rural residents and 134 (34.4%) mothers couldn’t read and write (Table 1).

**Table 1:** Maternal and child socio-demographic variables of common mental disorders in Gedio zone; 2022 (*N* = 389)

Variables	Category	Frequency (percentage)
Maternal age	Less than 20	26 (6.7%)
	20–24	43 (11.1)
	25–29	136 (35.0)
	30–34	123 (31.6)
	> 35	61 (15.7)
Religion	Protestant	288 (74)
	Orthodox Tewahedo	67 (17.2)
	Muslim	12 (3.1)
	Others	22 (5.7)
Marital status	Marred	311 (79.9)
	Divorced	34 (8.7)
	Died husband	24 (6.2)
	Other	20 (5.1)
Ethnicity	Gedio	348 (89.5)
	Oromo	26 (6.7)
	Amhara	12 (3.1)
	Others	3 (0.8)
Place	Wonago	118 (30.3)
	Yirgachefe	161 (41.4)
	Bule	110 (28.3)
Mother’s Educational status	1–8	154 (39.6)
	9–12	69 (17.7)
	diploma and above	32 (8.2)
	can't read and write	134 (34.4)
Husband educational status	1–8	158 (40.6)
	9–12	103 (26.5)
	diploma and above	41 (10.5)
	can't read and write	87 (22.4)
Residency	urban	61 (15.7)
	rural	328 (84.3)
Family size	Less than 5	160 (41.1)
	Greater or equal to 5	229 (58.9)

### Maternal related characteristics

Two hundred forty-five (63%) mothers were aged ≥ 18 years at marriage. Twenty (5.1%) of mothers had a history of sexual violence and 63 (16.2%) had chronic diseases. Seventy-five (19.3%) of participants took alcohol. Three hundred sixty-eight (94.6) of them had no physical violence. Two hundred ninety-two mothers (75.1%) had poor social support (Table 2).

### Children related characteristics

In this study, the median birth order was 3 (IQR: 2, 4). Two hundred fifty-one (64.5%) of mothers’ children aged less than 5 years and 221 (56.8%) were male. More than half (51.9%) of children were fully immunized. Only twenty-eight (7.2%) of mothers’ children had a chronic disease. Two hundred forty (61.7) of mothers gave birth at health facilities and 77 (19.8%) of mothers’ children had a previous history of malnutrition (Table 3).

**Table 2:** Maternal health status related variables of common mental disorders in Gedio zone; 2022 (*N* = 389)

Variables	Category	Frequency (percentage)
Age at marriage	Less than 18	144 (37)
	>=18	245 (63)
Mental depression in the family	Yes	43 (11.1)
	No	346 (88.9)
Maternal chronic disease	Yes	63 (16.2)
	No	326 (83.8)
Health sicking behavior	Yes	246 (63.2)
	No	143 (36.8)
Depression	Yes	172 (44.2)
	No	217 (55.8)
Sexual violence	Yes	20 (5.1)
	No	369 (94.9)
Smoking	Yes	9 (2.3)
	No	380 (97.7)
Chat chewing	Yes	11 (2.8)
	No	378 (97.2)
Alcohol drinking	Yes	75 (19.3)
	No	314 (80.7)
Physical violence	Yes	21 (5.4)
	No	368 (94.6)

**Table 3:** Children related variables who attend severe acute malnutrition treatment in Gedio zone, Southern Ethiopia, 2022 (*N* = 389)

Variables	Category	Frequency
Child age	Less than 5 year	251 (64.5)
	Five year	138 (35.5)
Child sex	Male	221 (56.8)
	Female	168 (43.2)
Immunization status	Not vaccinated	30 (7.7)
	Partial vaccinated	157 (40.4)
	Fully vaccinated	202 (51.9)
Other chronic disease	Yes	28 (7.2)
	No	361 (92.8)
Place of delivery	Health institution	240 (61.7)
	Home	149 (38.3)
History of malnutrition	Yes	77 (19.8)
	No	312 (80.2)

### Magnitude of common mental disorders

**Fig. 2:**
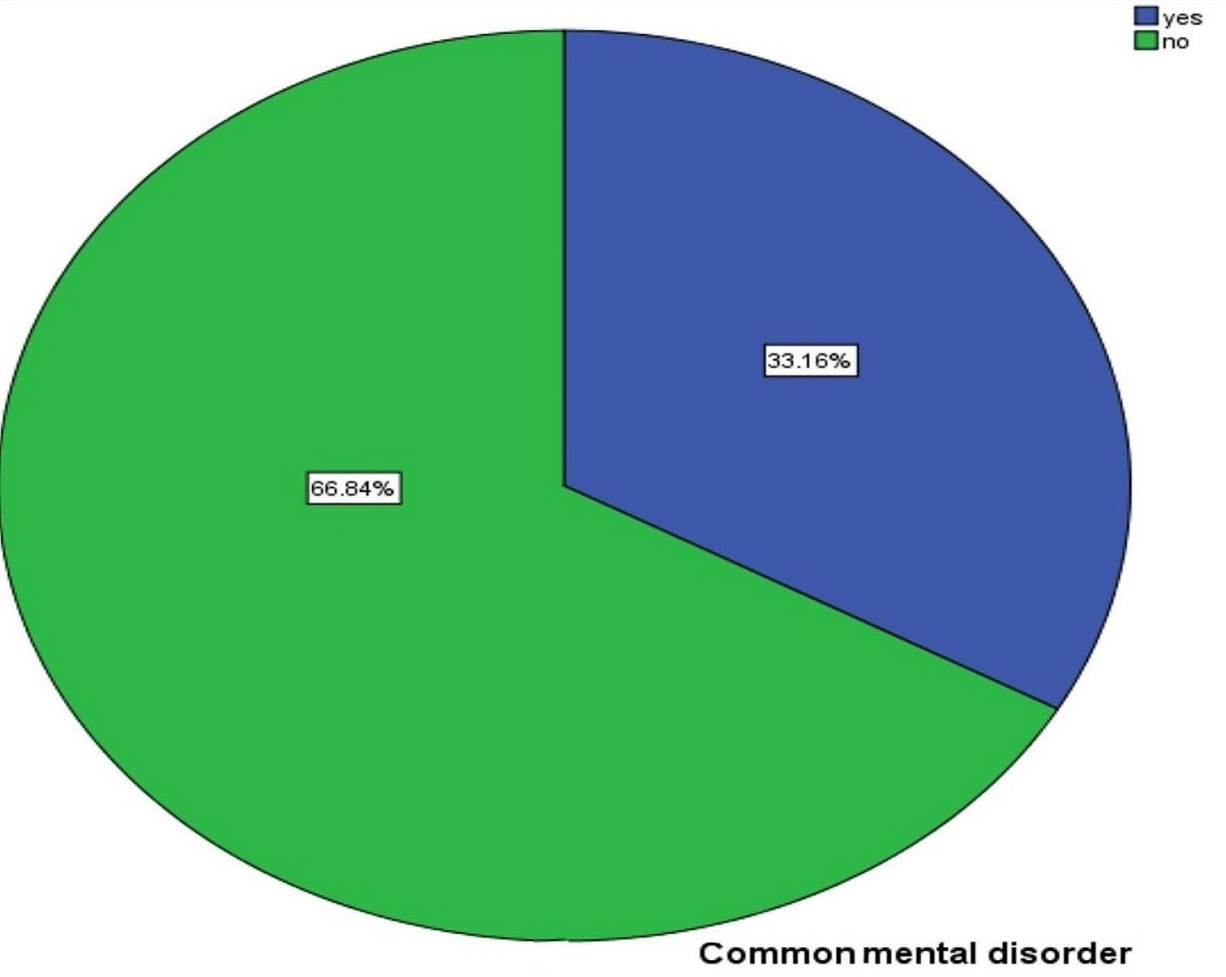
Magnitude of common mental disorders among mothers of children attending severe acute malnutrition treatment in Gedio zone, Southern Ethiopia, 2022 (*n* = 389)

In the present study, the magnitude of CMD among mothers of children on SAM treatment was 33.16% (95% CI [28.5–38]) (Fig. 2).

### Factors associated with common mental disorders

In the current study, logistic regression was employed. Model fitness was assessed by Hosmer and Lemeshow’s Test (p: 0.53). In bivariate analysis, only 11 variables had a p-value < 0.25 which were eligible for multivariable logistic regression. However, only six variables remained significantly associated with common mental disorders (social support, sexual violence, maternal chronic disorder, educational status, smoking, and children with another chronic disease) in multivariable logistic regression (Table 4).

**Table 4:** Bivariable and multivariable logistic regression for factors associated with Common mental disorders among mothers of children attending SAM treatment in Gedio Zone, Ethiopia, 2022 (*N* = 389)

Variables	Category	CMD		COR	AOR With 95% CI
		Yes	No		
Sex	Male	83	138	1.59 (1.03, 2.46)	2.00 (0.98, 3.20)
	Female	46	122	1	
Educational status (Mother’s)	can’t read and write	60	74	0.75 (0.40, 1.39)	1.95 (1.07, 3.55)**
	1–8	40	114	1.25 (0.50, 3.12)	1.19 (0.56, 2.55)
	9–12	22	47	0.43 (0.26, 0.71)	1.90 (0.59, 6.05)
	diploma and above	7	25	1	
Social support	Good	6	91	1	
	Poor	123	169	11.04 (4.68, 26.04)	14.0 (5.45, 35.9)**
Maternal chronic disease	Yes	38	25	3.92 (2.24, 6.87)	3.44 (1.72, 6.86)**
	No	91	235	1	
Cigarate smoking	Yes	6	3	4.18 (1.03, 16.99)	10.9 (1.78, 67.01)**
	No	123	257	1	
Alcohol drinking	Yes	34	41	1.91 (1.14, 3.20)	0.60 (0.31, 1.17)
	No	95	219	1	
Child with other chronic disease	Yes	14	14	2.12 (0.98, 4.63)	3.19 (1.13, 8.99)**
	No	115	246	1	
Maternal age	Less than 20	8	18	1	
	20–24	9	34	1.679 (0.55, 5.09)	1.99 (0.57, 6.92)
	25–29	42	94	0.99 (0.40, 2.17)	2.66 (0.89, 5.98)
	30–34	42	81	0.85 (0.34, 2.13)	0.68 (0.32, 0.98)
	> 35	28	33	0.52 (0.198, 1.38)	1.60 (0.75, 3.40)
Family size	Less than 5	46	114	1.4 (0.91, 2.12)	
	Greater or equal to 5	83	146	1	0.76 (0.42, 1.41)
Sexual violence	Yes	10	10	2.1 (0.85. 5.2)	4.14 (1.38, 12.4)**
	No	119	250	1	
History of malnutrition	Yes	37	40	2.21 (1.33, 3.68)	0.96 (0.51, 1.81)
	No	92	220	1	

Mothers who had poor social support had 14 [AOR: 14.0, 95% CI (5.45, 35.9)] times higher risk of CMD as compared to the opposite group. Participants who couldn’t read and write had 1.9 [AOR: 1.95, 95% CI (1.07. 3.55)] times more odds of CMD as compared to mothers whose education level was diploma and above. Furthermore, mothers who smoke cigarettes had 10.9 [AOR: 10.9, 95%CI (1.78, 67.01)] times more risk of CMD as compared to non-smokers (Table 4).

In this study, mothers of children with chronic disease had 3.2 times [AOR: 3.19, 95% CI (1.13, 8.99)] more risk of having CMD as compared to their opposite group. Moreover, mothers who had a history of sexual violence had 4 [AOR: 4.14, 95% CI (1.38, 12.4)] times more odds of CMD. Lastly, mothers with chronic disease had a 3.4 [AOR: 3.44, 95%CI (1.72, 6.86)] times higher burden to develop CMD in contrast to their counterparts (Table 4).

## Discussion

This study aimed to assess the magnitude of common mental disorders and associated factors among mothers of children attending SAM treatment in Gedio Zone, Southern Ethiopia. In this study, the magnitude of CMD was 33.16% (95% CI [28.5–38])) which was similar to a study done in Ethiopia (36.6%) [27]. It might be because of similar assessment tool was used to assess the dependent variable. However, it was high in contrast to another study conducted in Ethiopia among the general population (21.58%) [28]. This might be due to our study being conducted on the most vulnerable groups (mothers of SAM children).

Our finding was low as compared to studies conducted in Nigeria (40.7%) [5], Mali (71%) [17], Uganda (42%) [29] and Sudan 41.5% [30]. It might be due to that the previous studies were conducted with a small sample size and conducted on both programs but in our study, we considered only children on OTP. Furthermore, it might be because of socio-cultural differences.

Even though the problem is high in Africa, our finding is similar to studies done in Vietnam (31%) [31] and Brazil (34%) [32]. This similarity might be due to the above studies were also conducted in rural community in which maternal mental service is low. Our finding is high as compared to the global prevalence (17.6%) [33]. This might be due to socio-cultural and economic variation.

Mothers who had poor social support had a higher risk of developing CMD. This finding was supported by studies done in Ethiopia [27] and Brazil [34]. Women with poor social support might have feelings of neglect by others which lead to social isolation and emotional disturbances.

Illiterate mothers had more odds of CMD. This study was similar to studies conducted in Ethiopia [35], Tanzania [36] and Chile [37]. This similarity might be because education helps to improve cognitive ability. Moreover, higher education can enhance social capital, ultimately decreasing feelings of insecurity and vulnerability.

Mothers who smoke cigarettes have a higher risk of CMD as compared to nonsmokers. This study was similar to studies done in India [38] and Tanzania [39]. This similarity might be due to nicotine leading to poor concentration, low mood, and stress. Furthermore, smoking can increase the level of psychological distress and complicate or exacerbate mental illness and its treatment.

Mothers with chronic disease had more odds of CMD in contrast to their counterparts. This might be due to bidirectional relationship between chronic disease and common mental disorders and also due to those who are living with chronic illness having limited daily activities. As a result, they experience dissatisfaction in life which exposes them to emotional strains and ultimately to depression and anxiety.

Mothers of children with chronic diseases were more likely to have CMD. This might be due to the occurrence of additional chronic diseases on SAM children creating a high level of stress on mothers and making the level of mental illness double.

Lastly, mothers who had a history of sexual violence had higher odds of CMD. This finding is supported by a study done in India [38]. This could be due to sexual violence triggering feelings of helplessness, low self-esteem and depression and increasing the risk for mental disorders.

Generally, this study showed pertinent issues to overcome the problem. It will help clinicians and concerned bodies to address common mental disorders among mothers’ of malnourished children by recognizing the identified factors and putting focused intervention strategies towards the identified factors. Furthermore, it helps policy makers and program designers. However, our study didn’t show a causal relationship between the dependent and independent variables. Moreover, during sample size determination design effect were not considered.

## Conclusion

In this study, the magnitude of CMD was high. Six factors were significantly associated with common mental disorders social support, sexual violence, maternal chronic illness, educational status, smoking, and mother of a child with another chronic disease. The Federal Ministry of Education should incorporate maternal mental health assessment on severe acute malnutrition management guidelines. Community awareness regarding the effect of violence, substance use and social support on mental health also should be created by the local stakeholders. Furthermore, it is better to provide attention to mothers with chronic diseases and mothers of children with chronic illnesses.

## Data Availability

The data set analysed during the current study is available from the corresponding author upon reasonable request.

## Abbreviations

AHR: Adjusted Hazard Ratio
AOR: Adjusted Odds Ratio
CMD: common mental disorder
IQR: Inter quartile range
OR: Odd Ratio
SAM: severe acute malnutrition
SNNPR: South Nation Nationalities and People Region
WHO: World health organization

## References

[1] Organization WH. Depression and other common mental disorders: global health estimates. World Health Organization; 2017.

[2] Organization WH. Department of mental health and substance abuse. Promoting mental health: concepts, emerging evidence, practice: summary report Geneva. WHO, Department of Mental Health and Substance Abuse, Victorian Health Promotion Foundation (VicHealth), University of Melbourne; 2004.

[3] Ashaba S, Rukundo GZ, Beinempaka F, Ntaro M, LeBlanc JC (2015). Maternal depression and malnutrition in children in southwest Uganda: a case control study. BMC Public Health.

[4] Haithar S, Kuria M, Sheikh A, Kumar M, Vander Stoep A (2018). Maternal depression and child severe acute malnutrition: a case-control study from Kenya. BMC Pediatr.

[5] Abdullahi AT, Farouk ZL, Imam A (2021). Common mental disorders in mothers of children attending out-patient malnutrition clinics in rural north-western Nigeria: a cross-sectional study. BMC Public Health.

[6] Organization WH. The WHO special initiative for mental health (2019–2023): universal health coverage for mental health. JSTOR; 2019.

[7] Fisher J, Mello MCd, Patel V, Rahman A, Tran T, Holton S (2012). Prevalence and determinants of common perinatal mental disorders in women in low-and lower-middle-income countries: a systematic review. Bull World Health Organ.

[8] Klawetter S, McNitt C, Hoffman JA, Glaze K, Sward A, Frankel K (2020). Perinatal depression in low-income women: a literature review and innovative Screening Approach. Curr Psychiatry Rep.

[9] Dosani A, Arora H, Mazmudar S. mHealth and Perinatal Depression in Low-and Middle-Income countries: a scoping review of the literature. Int J Environ Res Public Health. 2020;17(20). 10.3390/ijerph17207679 PMC7589927 33096738

[10] Steel Z, Marnane C, Iranpour C, Chey T, Jackson JW, Patel V (2014). The global prevalence of common mental disorders: a systematic review and meta-analysis 1980–2013. Int J Epidemiol.

[11] Fisher J, de Mello MC, Izutsu T (2009). Mental health aspects of women’s reproductive health.

[12] Tronick E, Reck C (2009). Infants of depressed mothers. Harv Rev Psychiatry.

[13] Rayhan MI, Khan MSH (2006). Factors causing malnutrition among under five children in Bangladesh. Pak J Nutr.

[14] Black MM, Baqui AH, Zaman K, Arifeen SE, Black RE (2009). Maternal depressive symptoms and infant growth in rural Bangladesh. Am J Clin Nutr.

[15] Surkan PJ, Kennedy CE, Hurley KM, Black MM (2011). Maternal depression and early childhood growth in developing countries: systematic review and meta-analysis. Bull World Health Organ.

[16] Rahman A, Iqbal Z, Bunn J, Lovel H, Harrington R (2004). Impact of maternal depression on infant nutritional status and illness: a cohort study. Arch Gen Psychiatry.

[17] Stewart R, Bunn J, Vokhiwa M, Umar E, Kauye F, Tomenson B (2011). A prospective study of psychological distress among mothers of children admitted to a nutritional rehabilitation unit in Malawi. Child Care Health Dev.

[18] DE MIRANDA CT, TURECKI G, MARI JDJ, ANDREOLI SB, MARCOLIM MA (1996). Mental Health of the mothers of malnourished children. Int J Epidemiol.

[19] Firth JA, Motlhatlhedi K, Ganiyu AB, Setlhare V (2017). Association between depression in carers and malnutrition in children aged 6 months to 5 years. Afr J Prim Health Care Family Med.

[20] Pelletier DL, Frongillo EA, Schroeder DG, Habicht JP (1995). The effects of malnutrition on child mortality in developing countries. Bull World Health Organ.

[21] Patel V, Rahman A, Jacob K, Hughes M (2004). Effect of maternal mental health on infant growth in low income countries: new evidence from South Asia. BMJ.

[22] Chamois S, Golden M, Grellety Y. Ethiopia Protocol for the management of Severe Acute Malnutrition (2007). 2007.

[23] Ekwochi U, Asinobi NI, Osuorah CD, Ndu IK, Ifediora C, Amadi OF (2017). Incidence and predictors of mortality among newborns with perinatal asphyxia: a 4-year prospective study of newborns delivered in health care facilities in Enugu, South-East Nigeria. Clin Med Insights: Pediatr.

[24] Netsereab TB, Kifle MM, Tesfagiorgis RB, Habteab SG, Weldeabzgi YK, Tesfamariam OZ (2018). Validation of the WHO self-reporting questionnaire-20 (SRQ-20) item in primary health care settings in Eritrea. Int J Mental Health Syst.

[25] O. D. Social support-Consequences for individual and society. EUPHIX, EUphact Bilthoven: RIVM, EUphact\Determinants of health\Environment\Social support. 2009;;16.

[26] Humeniuk R, Ali R, Babor TF, Farrell M, Formigoni ML, Jittiwutikarn J (2008). Validation of the alcohol, smoking and substance involvement screening test (ASSIST). Addiction.

[27] Barsisa B, Derajew H, Haile K, Mesafint G, Shumet S (2021). Prevalence of common mental disorder and associated factors among mothers of under five year children at Arbaminch Town, South Ethiopia, 2019. PLoS ONE.

[28] Kassa GM, Abajobir AA (2018). Prevalence of common mental illnesses in Ethiopia: a systematic review and meta-analysis. Neurol Psychiatry Brain Res.

[29] Ashaba S, Rukundo GZ, Beinempaka F, Ntaro M, LeBlanc JC (2015). Maternal depression and malnutrition in children in southwest Uganda: a case control study. BMC Public Health.

[30] Mohammedahmed AS, Koko AEA, Arabi AM, Ibrahim MA (2020). Maternal depression, a hidden predictor for severe acute malnutrition in children aged 6–59 months: a case-control study at Omdurman Paediatrics Teaching Hospital, Sudan. Sudan J Paediatrics.

[31] Fisher J, Tran T, Nguyen TT, Nguyen H, Tran TD (2015). Common mental disorders among women, social circumstances and toddler growth in rural V ietnam: a population-based prospective study. Child Care Health Dev.

[32] Santos DS, Santos DN, de Cássia Ribeiro Silva R, Hasselmann MH, Barreto ML (2011). Maternal common mental disorders and malnutrition in children: a case–control study. Soc Psychiatry Psychiatr Epidemiol.

[33] Kassa GM, Abajobir A. Prevalence of common mental illnesses in Ethiopia: a systematic review and meta-analysis. Neurology, Psychiatry and Brain Research; 2018.

[34] Silva AG, Cerqueira AT, Lima MC (2014). Social support and common mental disorder among medical students. Revista brasileira de epidemiologia = Brazilian J Epidemiol.

[35] Woldetsadik AM, Ayele AN, Roba AE, Haile GF, Mubashir K (2019). Prevalence of common mental disorder and associated factors among pregnant women in South-East Ethiopia, 2017: a community based cross-sectional study. Reproductive Health.

[36] Paffer ATd, Ferreira HS, Cabral Júnior CR, Miranda CT (2012). Prevalence of common mental disorders in mothers in the semiarid region of Alagoas and its relationship with nutritional status. Sao Paulo Med J.

[37] Araya R, Montgomery A, Rojas G, Fritsch R, Solis J, Signorelli A (2018). Common mental disorders and the built environment in Santiago, Chile. Br J Psychiatry.

[38] Patel V, Xiao S, Chen H, Hanna F, Jotheeswaran AT, Luo D (2016). The magnitude of and health system responses to the mental health treatment gap in adults in India and China. Lancet (London England).

[39] Uriyo JG, Abubakar A, Swai M, Msuya SE, Stray-Pedersen B (2013). Prevalence and correlates of common mental disorders among mothers of young children in Kilimanjaro region of Tanzania. PLoS ONE.

